# Development of Polymeric Membranes Based on Quaternized Polysulfones for AMFC Applications

**DOI:** 10.3390/polym12020283

**Published:** 2020-02-01

**Authors:** Alessandra Carbone, Rolando Pedicini, Irene Gatto, Ada Saccà, Assunta Patti, Giovanni Bella, Massimiliano Cordaro

**Affiliations:** 1 CNR-ITAE Via S. Lucia sopra Contesse, 5, 98126 Messina, Italy; pedicini@itae.cnr.it (R.P.); gatto@itae.cnr.it (I.G.); sacca@itae.cnr.it (A.S.); patti@itae.cnr.it (A.P.); 2 Dipartimento di Fisica, Università della Calabria, Via Ponte P. Bucci, Cubo 31C, 87036 Arcavacata di Rende (CS), Italy; 3 Dipartimento di Scienze Chimiche, Biologiche, Farmaceutiche ed Ambientali, Università di Messina, V.le F. Stagno d’Alcontres 31, 98166 Messina, Italy; gbella@unime.it

**Keywords:** polysulfone, chloromethylation, quaternary ammonium, anion conductivity, AMFC

## Abstract

A series of quaternary ammonium-functionalized polysulfones were successfully synthesized using a chloromethylation two-step method. In particular, triethylammonium and trimethylammonium polysulfone derivatives with different functionalization degrees from 60% to 150% were investigated. NMR spectroscopic techniques were used to determine the degree of functionalization of the polymers. The possibility to predict the functionalization degree by a reaction tool based on a linear regression was highlighted. Anionic membranes with a good homogeneity of thickness were prepared using a doctor-blade casting method of functionalized polymers. The chemical–physical data showed that ion exchange capacity, water content, and wettability increase with the increase of functionalization degree. A higher wettability was found for membranes prepared by the trimethylamine (TMA) quaternary ammonium group. A degree of functionalization of 100% was chosen for an electrochemical test as the best compromise between chemical–physical properties and mechanical stability. From anionic conductivity measurement a better stability was found for the triethylamine (TEA)-based membrane due to a lower swelling effect. A power density of about 300 mW/cm^2^ for the TEA-based sample at 60 °C in a H_2_/O_2_ fuel cell was found.

## 1. Introduction

The production of electricity through alternative and environmentally friendly sources is undoubtedly a main goal of global research [1]. Among these, the technology of fuel cells has reached a high level of commercial competitiveness and, in combination with the search for new materials, provides a longer battery life [2,3]. Durable and recyclable materials and simple manufacturing methods are the key points on which the research is focused to improve the efficiency and reduce the cost of electricity per kWh [4]. The specific resistivity (ASR) of the cell components (electrolyte, anode, and cathode) must be low to ensure high power density and lower production costs per KW. Among the various types of developed fuel cells that require the use of hydrogen, alkaline fuel cells (AFCs) play a special role because they operate at lower temperatures compared to competitors, using non noble metals as electrocatalysts that reduce the cost of the system [5]. In particular, alkaline membrane fuel cells (AMFC) are widely studied because of the availment of a solid polymer instead of alkaline solution employed as an electrolyte in AFCs, in order to reduce the loss of performance due to the carbonate crystals precipitation and to prevent the corrosion of the system [6,7].

The conductivity depends on the number and kind of charged groups present on the polymeric species; therefore, the choice of the cationic group becomes of fundamental importance to having a correct anionic mobility. Anion exchange membranes (AEM) are designed to afford sufficient hydroxyl ions for ion exchange during electrochemical reactions in alkaline fuel cells [8,9,10,11,12].

Research has focused on the development of different polymers for this aim [2] and the most used are poly(arylene ethers) such as polysulfones, poly(ether ketones), poly(ether imides), poly(ether oxadiazoles), poly(phenylene oxides), polyphenylenes, etc. Among these polymers and cationic groups, polysulfone (PSF) and ammonium species can be good candidates for AEM preparation.

**Scheme 1:**
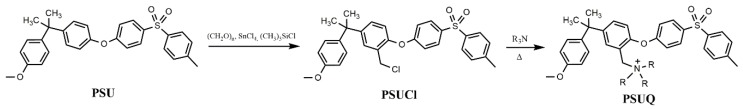
Classical method of functionalization of Polysulfone (PSU).

Polysulfone-based (PSU) polymers are thermoplastic stable materials characterized by their strength and stiffness and easy manufacturing of membranes with reproducible and controllable physical properties. The aromatic structure of this macromolecule is characterized by the presence of the sulfone groups (-SO_2_-) which gives the whole system an extraordinary resistance when exposed to mineral acids, alkaline conditions, electrolytes, and oxidants at a pH range from 2 to 13 [13]. The PSU is not an ionic conductor, so it must be functionalized by insertion of cationic or anionic groups through suitable synthetic steps. In order to insert cationic functionalities to the PSU backbone, the synthesis of trialkylammonium polysulfones via S_N_2 reactions on chloromethylated derivatives is usually carried out (Scheme 1) [10].

AEM of PSU are formed with fixed cationic groups and counter anions, generally hydroxide ions that sustain the conductivity. The cationic head-group chemistries that have been studied consist of ammonium, imidazolium, guanidinium, triazole, phosphonium, and sulfonium cations, amongst others [2]. However, among these, only ammonium groups present sufficient stability for use in an aggressive environment and quaternary ammonium based on trimethylamine is by far the most widely studied due to its sufficient stability in an alkaline environment.

Despite the wide literature existing on polysulfone-based membranes, few papers report the fuel cell characterization and, where reported, the performance does not exceed 250 mW/cm^2^ in a single H_2_/O_2_ fuel cell using Pt as an electrocatalyst in both anode and cathode [4].

In this paper, the development and characterization of quaternized polysulfone was carried out. The results highlight the novelty of the possibility to predict the functionalization degree by a reaction tool based on a linear regression. Moreover, a surprisingly higher stability to the alkaline environment and temperature for triethylamine than trimethylamine was found, as generally reported in the literature. In addition, fuel cell tests carried out in a 25 cm^2^ single cell using H_2_/O_2_ as reactant gases and Pt as an electrocatalyst in both anode and cathode reached a maximum power density of about 300 mW/cm^2^, higher than those reported in several papers.

## 2. Materials and Methods

### 2.1. Polymer Synthesis and Membrane Preparation

#### 2.1.1. Polymer Functionalization

**Scheme 2:**
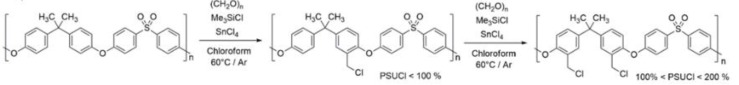
Schematic representation of the chloromethylation procedure of PSU.

The chloromethylated PSU derivatives were synthesized from commercial PSU (*M*_n_ ~ 22,000) with paraformaldehyde in an equimolar amount of chlorotrimethylsilane (Me_3_SiCl) and stannic tetrachloride (SnCl_4_). The reaction was carried out in anhydrous chloroform under an N_2_ atmosphere and the mixture was refluxed at 60 °C for a variable time according to the desired derivatization percentage (Scheme 2).

**Scheme 3:**
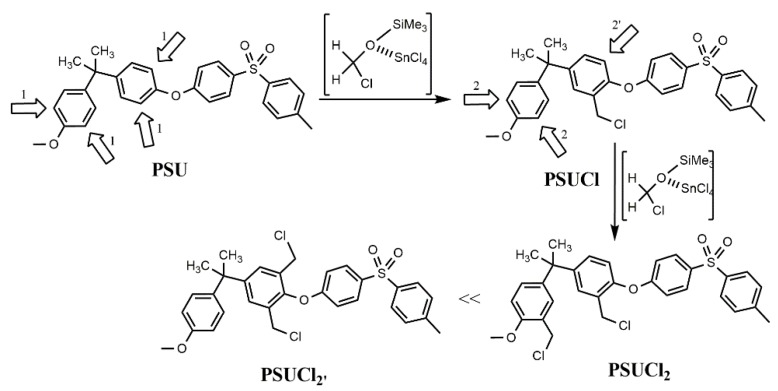
Chloromethylation mechanism of PSU.

The chloromethylation reaction occurs through an electrophilic aromatic substitution mechanism, as well known [14], favored by the presence of tin chloride (Lewis acid) which activates the reaction intermediate (Scheme 3).

The first approach of the –CH_2_Cl group to the *ortho* positions to the ether oxygen {1} is equally favored. Instead, the approach of the second –CH_2_Cl group to the *ortho* positions of the adjacent phenol {2} is preferred with respect to the remaining *ortho* position of the same ring {2′}, due to steric hindrance.

Chloromethylation products were synthesized with a degree of functionalization from 60% to 150%. Therefore, the optimization of reaction parameters (stoichiometry, temperature, and time) were analyzed. The temperature was kept constant to avoid the formation of polyalkylated and cross-linked side-products. The anhydrous solvent and inert atmosphere have proved to be fundamental to preserve over time the formation of reactive intermediate [15].

As well known [16], the increase in temperature accelerates the chloromethylation reaction; unfortunately at values above 60 °C gelation occurs and side cross-links are formed, effects that worsen the ability of the membrane to transport ions. We believe that it is possible to select the degree of functionalization of the polymer, keeping the temperature at a 55–60 °C constant and monitoring only the time. The necessary condition to favor the course of the reaction is the presence of the Lewis acid intermediate (Scheme 3); this was achieved by increasing the quantity of reagents and maintaining the inert atmosphere. Two reaction conditions were used:

Method A: PSU (1 eq):(CH_2_O)_n_ (10 eq):((CH_3_)_3_SiCl) (10 eq):SnCl_4_ (0.1 eq)—Temperature 60 °C
Method B: PSU (1 eq):(CH_2_O)_n_ (20 eq):((CH_3_)_3_SiCl) (20 eq):SnCl_4_ (0.1 eq)—Temperature 55 °C

The method A allowed to obtain derived PSUs with a degree of chloromethylation less than 70%, minimizing the use of harmful reagents and reducing costs. The method B, which is used for the synthesis of derived PSUs with a chloromethylation degree higher than 70%, requires a larger amount of reagents, and in this way it was possible to obtain PSUs with up to 150% functionalization (Table 1). All products were analyzed by ^1^H-NMR using CDCl_3_ as a solvent.

Method A: PSU (3.7 g, 8.55 mmol) and paraformaldehyde (2.6 g, 85.5 mmol) were stirred in anhydrous chloroform (410 mL) in a two-necked flask in an argon atmosphere. After the complete dissolution, trimethylsilyl chloride (10.8 mL, 85.5 mmol) and SnCl_4_ (0.1 mL, 0.85 mmol) were slowly added dropwise. The reaction was maintained under stirring in a thermostatic bath at 60 °C in the argon atmosphere. The mixture was transferred to methanol (400 mL) and was allowed to settle at room temperature for a few hours. The white solid obtained was filtered with a Büchner set-up and washed several times with water and finally dried under vacuum for 24 h at 60 °C.

Method B. PSU (5.0 g, 11.1 mmol) and paraformaldehyde (6.8 g, 226 mmol) were stirred in anhydrous chloroform (250 mL) in a two-necked flask in an argon atmosphere. After the complete dissolution, trimethylsilyl chloride (28.7 mL, 226 mmol) and SnCl_4_ (0.13 mL, 1.1 mmol) were slowly added dropwise. The reaction was maintained under stirring in a thermostatic bath at 55 °C in the argon atmosphere. The work-up of the reaction was identical to method A.

#### 2.1.2. General Method of Amination of PSUCl

The quaternization derivatives (PSUQ) were obtained by treatment of chloromethylation derivative (PSUCl and PSUCl_2_) with an excess of trialkyl amine in dimethyl sulfoxide (DMSO) at 50 °C for 48 h.

PSU with a different chloromethylation degree (1 eq) was dissolved in DMSO by stirring at 40 °C for 1 h in an argon atmosphere. Then pure trialkyl amine (10 eq) was added and the solution was kept under stirring for 48 h at 50 °C, after which the solvent was removed under vacuum using rotavapor at 80 °C.

**Scheme 4:**
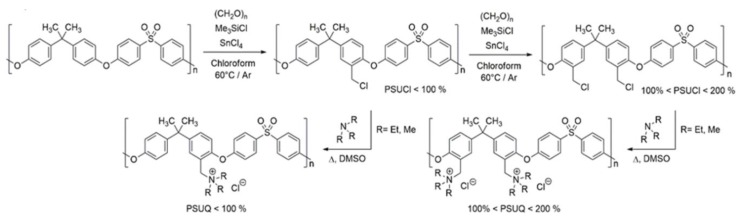
Detailed description of PSU functionalization.

Two different amines were used, in particular trimethylamine (TMA) and triethylamine (TEA), in order to understand their influence on the final membrane properties (Scheme 4).

#### 2.1.3. Membrane Preparation

Polysulfone-based membranes were prepared by dissolving the polymers in DMSO at 80 °C. In order to obtain a suitable viscosity a concentration step was performed, allowing the process of stratification by means a doctor-blade method. The cast membrane was left to dry at a standardized temperature of 80 °C for 3 h [17]. The same procedure was used for different functionalization degrees and quaternary ammonium groups. The dried membranes were first immersed in NaCl 1 M solution for 72 h at room temperature and afterwards in KOH 1 M solution before the electrochemical tests. The prepared membranes are listed in Table 2.

#### 2.1.4. Electrodes Preparation

The ionomer dispersion was prepared by solubilizing the commercial Fumion^®^ FAA3-shredded film solid polymer (FUMATECH BWT GmbH) in an alcoholic solution of n-propanol and ethanol (1:1 wt) in order to have a ~5 wt % dispersion.

The electrodes were prepared using a spray technique as described elsewhere [17]. In particular, the catalytic ink was obtained by mixing the commercial 40 wt % Platinum on a carbon (Pt/C) electro-catalyst (Alfa Aesar) and the FAA3 ionomer (20 wt % of the total solid content) (FUMATECH BWT GmbH). A Platinum (Pt) loading of 0.5 mg/cm^2^ both for the anode and the cathode side was used. The catalytic ink was sprayed onto the Sigracet 25-BC Gas Diffusion Layer (SGL). The prepared electrodes were kept at 60 °C for 30 min to allow the solvent evaporation.

### 2.2. Characterizations

#### 2.2.1. NMR Spectroscopy

The degree of chloromethylation of the polysulfone was investigated by nuclear magnetic resonance (NMR) spectroscopy. Proton NMR (^1^H-NMR) spectra were obtained on a Varian-500 NMR (Palo Alto, CA, USA) using CDCl_3_ and d_6_-DMSO as solvents for the analysis of chloromethylated and quaternized derivatives, respectively. The IR analyzes were performed on a Bruker spectrometer. The PSU used in pellets was commercial. Commercial anhydrous chloroform was used. Chemical reagents were used without further purification.

#### 2.2.2. Thickness Measurements and Ion Exchange Capacity

The thickness of membranes was measured on samples equilibrated at room temperature and humidity with a thickness gauge (Mitutoyo Italiana s.r.l. Mod. ID-C112PB, MilanItaly). The thickness reported in Table 2 is an average value of about 20 points.

The total amount of ionic groups contained in the membrane was determined by a back-titration based on the Volhard method using samples with Cl– as a counter-ion, after an exchange in NaCl 1 M solution (72 h).

The dried membranes were immersed in a NaNO_3_ 0.1 M solution for 48 h at room temperature, and then it was removed. To the solution 5 mL of AgNO_3_ 0.1 M and 5 drops of Fe(NO_3_)_3_ (11 wt %) were added. A back-titration of the solution with KSCN 0.1M was performed. The amount of volume of the KSCN needed to achieve the so-called equivalent point (V_KSCN_ e.p.) was determined.

The IEC was calculated as follows:

IEC = (V_AgNO3_ − V_KSCN_ e.p.) [KSCN]/m_dry_

where:

V_A_g_NO3_ is the initial volume, expressed in mL
V_KSCN_ e.p is the volume of the titrant at the equivalent point expressed in mL
[KSCN] is the concentration of titrant, expressed in molarity
m_dry_ is the dry mass of the sample determined at 50 °C for 2 h under vacuum (10 mbar), expressed in grams.

#### 2.2.3. Thermogravimetric Analysis

Thermo gravimetric analysis (TGA) of the polymer electrolyte was performed using a thermo balance Netzsch (mod. STA 409, Selb, Germany) in static air by monitoring the percentage of mass loss change in the temperature range 20–1200 °C, using a scan rate of 5 °C/min.

#### 2.2.4. Contact Angle Measurements

Contact Angle measurements were performed on membrane samples (3 × 3 cm^2^), with Cl^−^ as a counterion. Five different areas were analyzed to determine the average value. The static contact angle measurements were carried out using the OCA-25 model of the DataPhysics Instruments GmbH (Filderstadt, Germany) following a sessile drop procedure and employing water as a liquid with a volume drop of 2 μL dispensed with a drop rate of 0.5 μL/s on the surface of the films to be studied. The drop-shape was analyzed by an ellipse fitting method.

#### 2.2.5. Anion Conductivity

The in-plane anion conductivity of samples with OH^−^ as a counterion was carried out using a four-electrodes method and measured by electrochemical impedance spectroscopy (EIS). The EIS parameters were: 100 KHz–1 Hz of frequency range and a 50 mV of amplitude. The measurements were performed in the temperature range 30–80 °C, fluxing humidified N_2_ (100% RH) at atmospheric pressure.

#### 2.2.6. MEAs Preparation and Fuel Cell Tests

Membrane electrodes assemblies (MEAs) were obtained by assembling the electrodes and the membrane directly in the 25 cm^2^ single cell hardware, after the ion exchange in a KOH 1 M solution at room temperature for 24 h.

Fuel cell tests were carried out at 60 °C, with fully humidified H_2_ and O_2_ as reactant gases, at atmospheric pressure, with a stoichiometry of 1.5 and 2 at 1 A/cm^2^ for H_2_ and O_2_, respectively.

The cell was activated at 60 °C for 1 h by operating at 0.5 V before the polarization curves to have a MEA conditioning and to purge the CO_2_ present in the environment [18,19].

## 3. Results

### 3.1. NMR Results

**Scheme 5:**
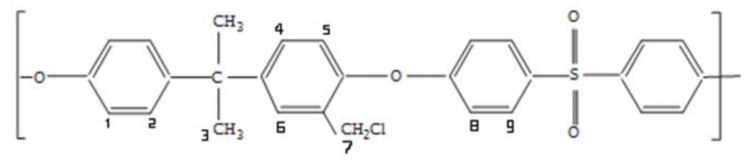
Chloromethylated polysulfone.

The chemical shifts of the PSU were found as follows (CDCl_3_, ppm): 6.90–7.86 (aromatic hydrogens) and 1.72 (CH_3_). As reference for all chloromethylated PSUs, the chemical shifts of PSU100 are listed below (CDCl_3_, ppm): 7.86 (m, 2 H); 7.36 (s, 1 H); 7.23 (d, 1 H); 7.16 (d, 1 H); 7.01 (m, 2 H); 6.94 (d, 1 H); 6.84 (d, 1 H); 4.54 (s, 2 H); 1.71 (s, 3 H); 1.70 (s, 3 H). The characteristic chemical shift at 4.54 confirmed the formation of the chloromethylated polysulfone, as reported in Scheme 5. The percentage of functionalization was calculated from the ratio between the integral areas of the peaks inherent to the –CH_2_Cl hydrogens (δ = 4.54 ppm) and the *ortho*-hydrogens of sulfonic-phenyl (δ = 7.86 ppm).

**Figure 1:**
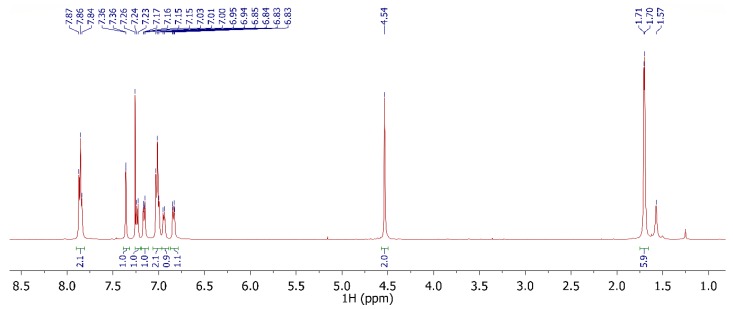
^1^H-NMR of 100% chloromethylated PSU (PSU100) in CDCl_3_.

**Figure 2:**
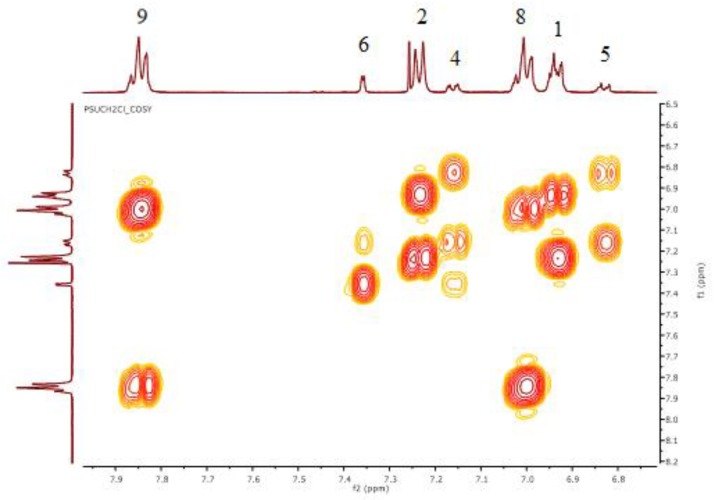
COSY–NMR of 50% chloromethylated PSU.

The assignment of the proton’s correlations of the PSUCl was carried out by two-dimensional COSY analysis (Figure 1 and Figure 2).

The study of derivatization over time, starting from the 65% derivative PSUCl, showed an increasing linear trend that allowed us to obtain any derivative with a degree of functionalization up to the 150% desired (see Supplementary Materials Figure S1). A regression wizard was carried out to find a curve which matches a series of experimental data points.

**Figure 3:**
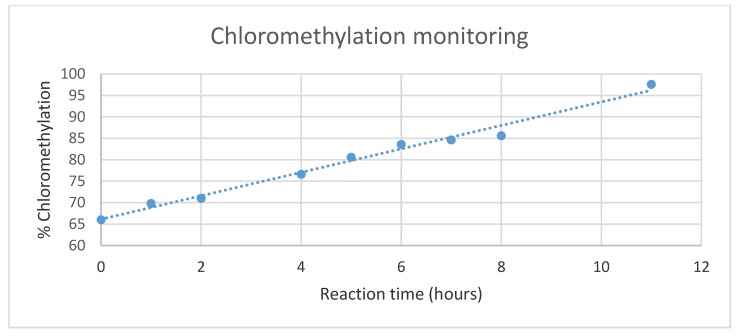
Chloromethylation degree variation versus reaction time.

The curve-fitting allowed us to visualize and plot the curve that best describes the shape and behavior of percentage of chloromethylation as a function of time (Figure 3). The best fitting was found with a linear regression. Below is the function used: *y* = 2.7354*x* + 66.12 R^2^ = 0.9852.

The assignment of the proton’s correlations of the PSUQ was carried out by two-dimensional COSY analysis (see Supplementary Materials Figures S2 and S3).

### 3.2. Ion Exchange Capacity

The ion exchange capacity of the membranes (Table 3) containing chloride as a counter-ion revealed an increase of exchangeable ions proportionally to the degree of functionalization. A slight difference can be found between the two different quaternary ammonium groups due to the steric hindrance of TEA in respect to TMA, which is probably a bit less accessible from all the ions. The data are in agreement with NMR results.

### 3.3. Thermal Stability of PSU

**Figure 4:**
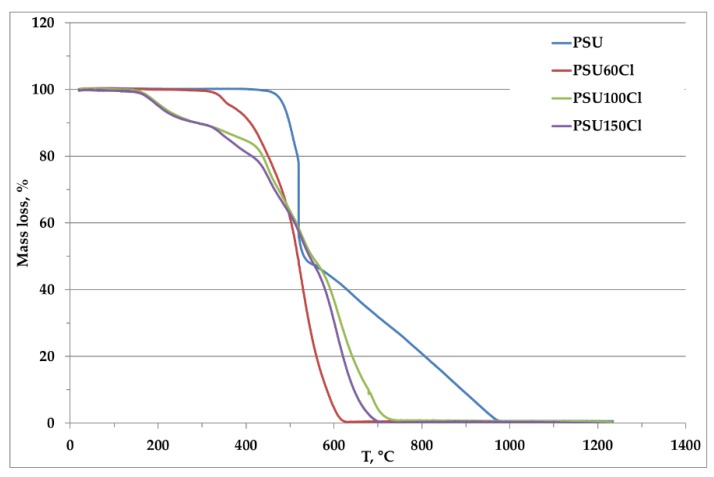
Comparison of thermograms of chloromethylated samples and pristine PSU.

The thermal analysis was carried out on samples with chloride as a counterion, in order to verify the thermal stability in the range of interest for the real applications. In addition, it is possible to check how the chloromethylation and quaternarization reactions can affect the polymer thermal stability. In Figure 4 the comparison of the pristine polymer with the chloromethylated polymer is reported for each functionalization degree.

It is evident that the chloromethylation modified the thermal profile of the PSU. In fact, in PSU a trend towards a plateau from room temparature up to 450 °C was observed, meaning that any thermal process occurred before the decomposition temperature of the polymer. Regarding the chloromethylated samples, it was evident that a mass loss due to functional groups decomposition occurred at temperatures lower than 450 °C. In particular, this temperature decresed with the increase of the degree of functionalization, changing from about 330 °C for PSU60 to about 150 °C for samples PSU100 and PSU150. The decomposition temperature of the polymer backbone was also shifted to a lower temperature than the pristine PSU (Table 3). Also in this case the decomposition temperature was reduced with the introduction of the functional groups.

In Table S1, a water loss of the samples calculated from room temperature up to about 150 °C is reported. The samples were washed in water at 30 °C and left to equilibrate at room humidity.

The water uptake is an important parameter that is related to the ionic conductivity and, consequently, to the fuel cell performance [20,21,22]. These data can be considered as the water uptake at room conditions (temperature and humidity) and are proportional to the functionalization degree. In fact, the water content increases with the increase of chloromethylation and remains quite unaltered when TEA or TMA was used. The data reported are below 12% because the counter-ion is Cl^−^ and the affinity to water is, obviously, lower than hydroxide. In addition, the measurements were carried out after room equilibration and not after immersion in liquid water, but the trend is strongly indicative of the water content as a function of the functionalization degree.

In Table S1 are also reported, as a function of the chloromethylation degree and quaternarization with two different amines, the temperature attributed to the loss of functional groups (*T*_fg_) and decomposition (*T*_d_) of the polymer backbone.

As above mentioned, the increase of functionalization degree produces a reduction of both T_fg_ and T_d_, and the reduction is more pronounced when triethylamine for the quaternarization process is used. The thermal stability of polymers produced using TMA as a quaternarising agent possesses a higher thermal stability than other amines, in accordance with literature data [23]. For sample PSU60 a strong reduction converting the chloromethylated into trimethyl quaternary ammonium and successively to a triethyl one was detected. This difference was mitigated further when samples PSU100 and PSU150 were analyzed. In any case, the PSU100 membrane seems to be less affected from the preparation steps of the quaternarization, making it more stable to the conversion.

### 3.4. Wettability of PSU

**Figure 5:**
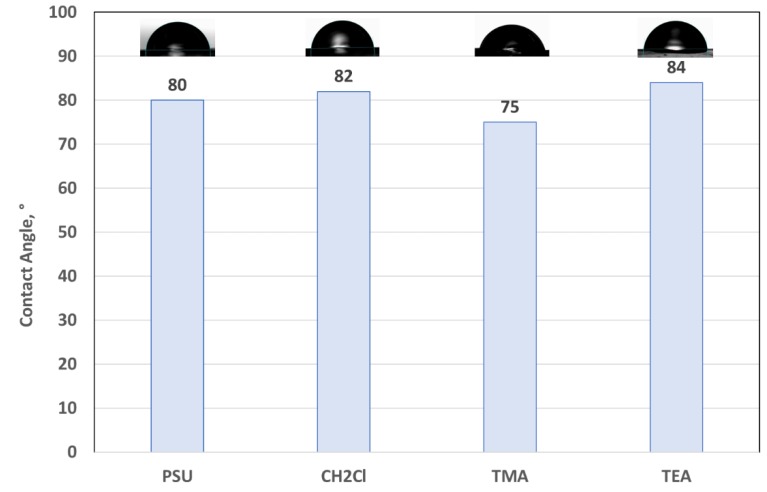
Contact angle of 60% DF samples as a function of different functionalization reaction steps. The drop used for the contact angle calculation is reported above each sample.

The surface properties of membranes can be characterized by measuring the contact angle. The characteristics of hydrophilicity or hydrophobicity of membranes can be determined and a low contact angle indicates high water affinity [24]. In Figure 5 the influence of the functionalization reaction steps on the wettability properties of membranes is reported. Only the comparison of the sample with 60% DF is reported, but the same trend was found for the other samples. All the investigated samples mainly presented hydrophilic characteristics because the measured values of the contact angles were lower than 90° [25]. The quaternized samples were exchanged in NaCl 1M before the measurements in order to have them in a completely chloride form.

It is evident that the introduction of the chloromethyl group does not substantially change the surface properties of the membrane, causing only a slight increase in the contact angle due to the presence of the functional group [26]. The insertion of the trimethylamine reduces the contact angle value from 82° down to 75°, due to the pronounced hydrophilic property of the amine, while the insertion of TEA increases the value from 82° up to 84°, due to a less hydrophilic property of this group in respect to the TMA. The reported data are an average value of 5 measurements and can be considered as a trend.

### 3.5. Conductivity of PSU

**Figure 6:**
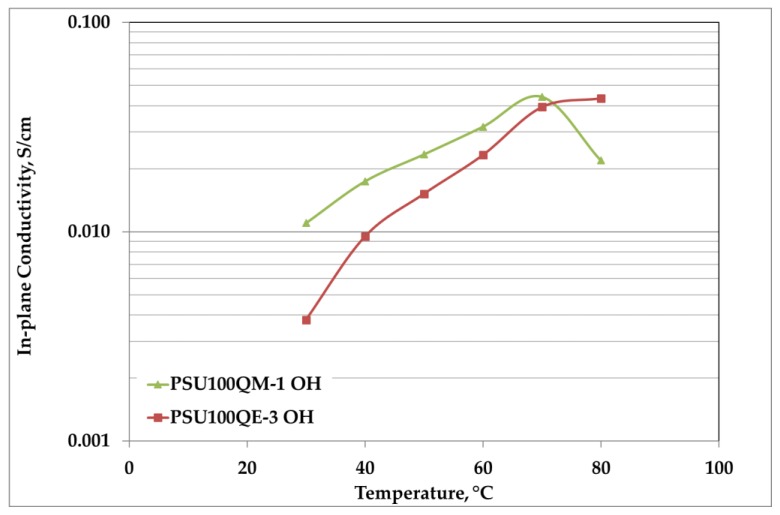
Anion conductivity (Cl^−^) of 100% DF samples with different quaternary ammonium groups.

The electrochemical characterization in terms of anion conductivity was evaluated only for samples having a 100% degree of functionalization and TMA or TEA as quaternary ammonium (Figure 6). The samples were tested after the exchange in a KOH 1 M solution for 24 h, so as to have them in hydroxide form, in order to reproduce the same samples used in fuel cell tests. It was not possible to test the other samples due to their poor mechanical properties after the alkaline exchange.

As expected, the increase in conductivity is proportional to the temperature. The maximum value of 44 mS/cm for PSU100QM-1, against 39.5 mS/cm of the PSU100QE-3 membrane, was reached at 70 °C. The sample PSU100QM-1 presented the highest values from 30 to 70 °C, and after this temperature a drop of conductivity was detected due to the swelling effect that causes a collapse of the mechanical properties. By contrast, the PSU100QE-3 sample, below 70 °C, had lower conductivity values than the PSU100QM-1, but steadily increased over the entire temperature range studied. This behavior can be justified by a lower swelling effect of the quaternary polymer with TEA, in accordance with the above-mentioned properties of wettability. In fact, the membrane that had a more hydrophilic behavior (PSU100QM-1) showed the highest swelling effect.

It is assumed that the hydroxide transport mechanism is the combination of Grotthus and vehicular conduction theories under humidified conditions [27]. The activation energy value reported for this mixed mechanism, with prevalence of the vehicular one, is between 10–15 kJ/mol [28]. For the OH^−^ conductivity of the polymers, in the temperature range 30–80 °C, linearity to the Arrhenius was experimentally observed. Approximate values of activation energy (30.3 kJ/mol for PSU100QM-1 and 44.6 kJ/mol for PSU100QE-3) were extrapolated. Thus, the higher calculated values suggest a predominance of the Grotthus mechanism with respect to the mixed conduction mechanism [6].

This can be explained considering that the hydroxide ions tend to move through a hydrogen-bond network [9].

### 3.6. Fuel Cell Tests

**Figure 7:**
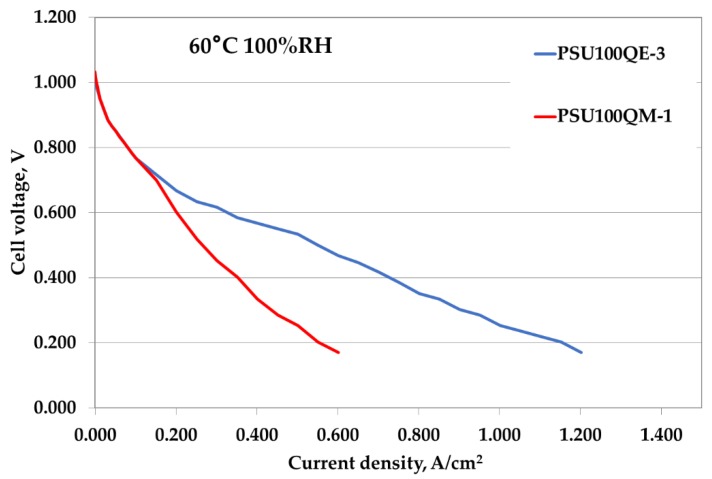
Polarization curves comparison of PSU-based membrane electrodes assemblies (MEAs).

The electrochemical performance in terms of polarization curves was evaluated only for samples having a 100% degree of functionalization and TMA or TEA as quaternary ammonium due to the degradation of the other samples after the alkaline treatment. Fuel cell tests in a 25 cm^2^ single cell were carried out at 60 °C, as reported in Figure 7. The performance of the TEA-based membrane was higher than TMA-based sample, with a limiting current (1.2 A/cm^2^) approaching the double of the value of TMA (0.6 A/cm^2^). Even if the performance seems to be in contrast with the conductivity data, this behavior can be explained considering that the two characterization methods affect different sections of the membrane. In the conductivity, the in-plane section was analyzed, while in the fuel cell test the through plane one was analyzed. When an anisotropic arrangement of the polymer occurs, different results between the two techniques can be obtained. It must be considered that the PSU100QM-1 does not have optimal mechanical properties and consequently exhibits a less efficient performance; this is due to a more pronounced swelling effect.

As reported in Table 4, both the cell resistance and the open circuit voltage (OCV) are comparable between the two samples and the maximum power density of PSU100QE-3 is about two times the value of PSU100QM-1.

More in detail, the OCV values are in agreement with data reported in the literature for polyaromatic membranes, meaning that the reactant gas crossover is limited and no drop of cell potential can be detected. The membranes preparation is based on an in-lab standardized procedure and a temperature of 80 °C is used, but the effect of the membranes’ drying temperature or thermal annealing as a post-casting treatment on the fuel cell performance is a key point. Hence, a further study on the influence of the thermal procedure can be useful to improving performance, since the gas permeability depends on the pre-treating procedure. It has been clearly demonstrated [29,30,31] that the thermal pre-treatment reduces the gas crossover, and this is important in particular regarding the CO_2_ sorption that drastically reduces the fuel cell performance.

The data reported in this paper can be considered as preliminary results and further investigation can be performed in order to evaluate the influence of the quaternary ammonium groups on the fuel cell performance, and in particular on the long term stability.

## 4. Conclusions

In the present study, chloromethylated polysulfone was obtained and reaction parameters were optimized to predict the functionalization percentage. A reaction tool based on a linear regression was set for this purpose. Starting from the synthesized polymers, the corresponding membranes were prepared. The influence of two different quaternary ammonium groups on the membrane properties, in particular TMA and TEA, were evaluated, and a higher stability to an alkaline environment and temperature for TEA than TMA was found. The thermal stability of PSU with the insertion of CH_2_Cl and quaternary ammonium groups was progressively reduced. The properties of hydrophilicity also depend on the used quaternary ammonium groups. In fact the TEA-based samples showed a greater tendency to hydrophobicity compared to the TMA-based samples. A degree of functionalization of 100%, as the best compromise between physical–chemical properties and mechanical stability, was chosen for the electrochemical characterizations. The TMA-based sample, with a higher ion exchange capacity and hydrophilicity, showed a higher anion conductivity than TEA-based one with a maximum value of 44 mS/cm against 39.5 mS/cm at 70 °C, respectively. The activation energies calculated for both membranes suggest a predominance of the Grotthus conduction mechanism. On the contrary, a better performance for TEA-based sample in AMFC configuration at 60 °C was found, reaching a maximum power density of about 300 mW/cm^2^, due to the limited swelling phenomenon. Hence, taking into account the chemical–physical and electrochemical results of different quaternary ammonium-based membranes, the PSU100QE-3 can be considered as the best compromise and it seems to be the most promising for fuel cell applications.

## Figures and Tables

**Table 1:** Degree of functionalization (DF) of chloromethylation PSU.

Polymer	Method	Concentration, eq	T, °C	Time, h	DF, %
PSU60	A	1:10:10:0.1	60 °C	24	60
PSU100	B	1:20:20:0.1	55 °C	24	100
PSU150	B	1:20:20:0.1	55 °C	48	150

**Table 2:** Prepared membranes.

MEMBRANE	Q	Thickness, μm	Manufactured Properties
PSU60QE-1	TEA	32	Flat, homogeneous, robust
PSU60QM-2	TMA	49	Brittle
PSU100QE-3	TEA	43	Flat, homogeneous, robust
PSU100QM-1	TMA	48	Flat, homogeneous, robust
PSU150QE-2	TEA	45	Not homogeneous, robust
PSU150QM-1	TMA	47	Not homogeneous, robust

PSUN_x_Q_y_-z: N_x_ is % of functionalization, Q_y_ is E = TEA and M = TMA, z = number of batch preparation.

**Table 3:** IEC of quaternized PSU membranes.

Samples	% CH_2_Cl	IEC TMA, meq/g	IEC TEA, meq/g
PSU60	60	-	1.50
PSU100	100	1.71	1.67
PSU150	150	2.02	1.99

**Table 4:** Electrochemical parameters of the tested MEAs. OCV: open circuit voltage.

MEMBRANE	R_cell_, Ωcm^2^	OCV, V	PD, mW/cm^2^
PSU100QE-3	0.157	1.015	293
PSU100QM-1	0.161	1.032	142

## Supplementary Materials

The following are available online at https://www.mdpi.com/2073-4360/12/2/283/s1: kinetic study of chloromethylation of PSU by NMR; 2D-COSY of trialkylammonium PSU; Table of thermal decomposition of polymers; Figure S1: Superimposed 1H-NMR of the kinetic study of chloromethylation of PSU from 65% to 100%; Figure S2: COSY-PSU100QE; Figure S3: 2D-COSY-NMR PSU100QM; Table S1: water uptake, temperature of defunctionalization, and decomposition of chloromethylated and quaternarized samples.
