# Supplementary material for: Development of Polymeric Membranes Based on Quaternized Polysulfones for AMFC Applications
Source: Polymers (Basel). 2020 Feb 1;12(2):283. doi: 10.3390/polym12020283 (PMC7077470; doi:10.3390/polym12020283)
Supplement: Supplementary file 1 [file polymers-12-00283-s001.pdf]

Supplementary Materials

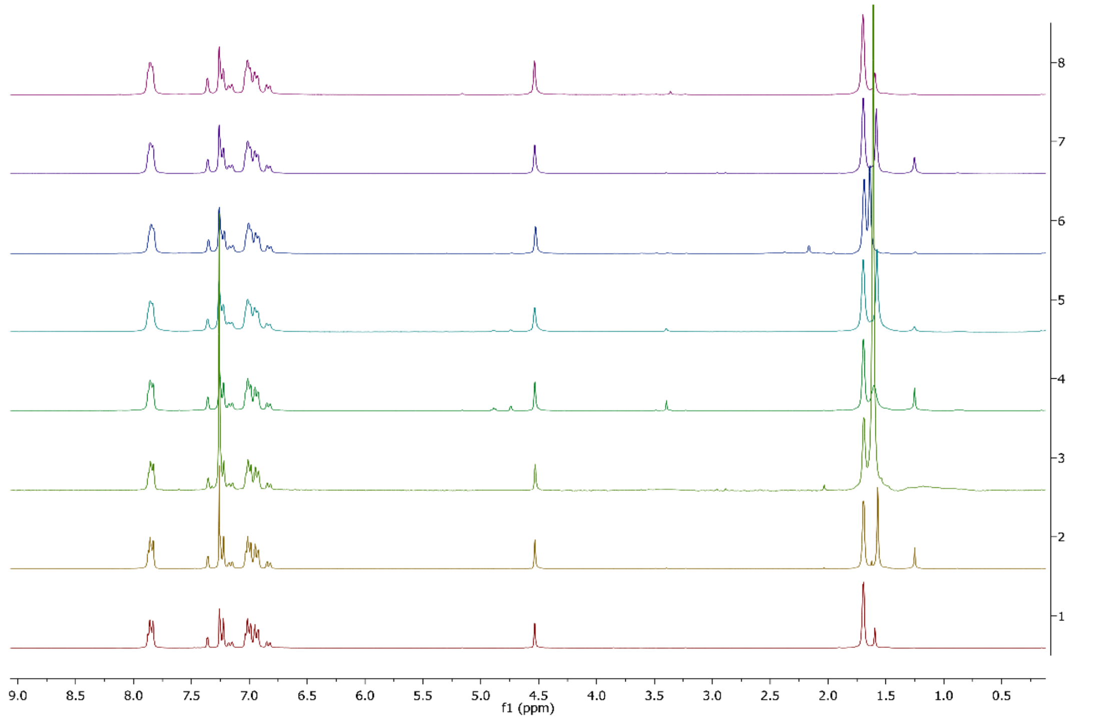

Figure S1 – Superimposed 1H-NMR of the kinetic study of chloromethylation of PSU from 65% to 100 %.

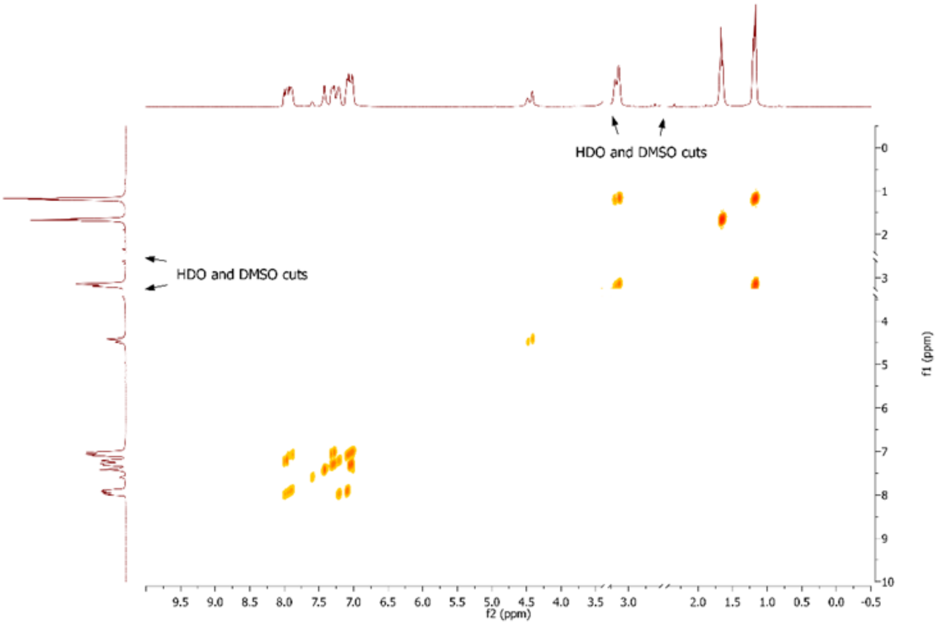

Figure SI2 COSY-PSU100QE

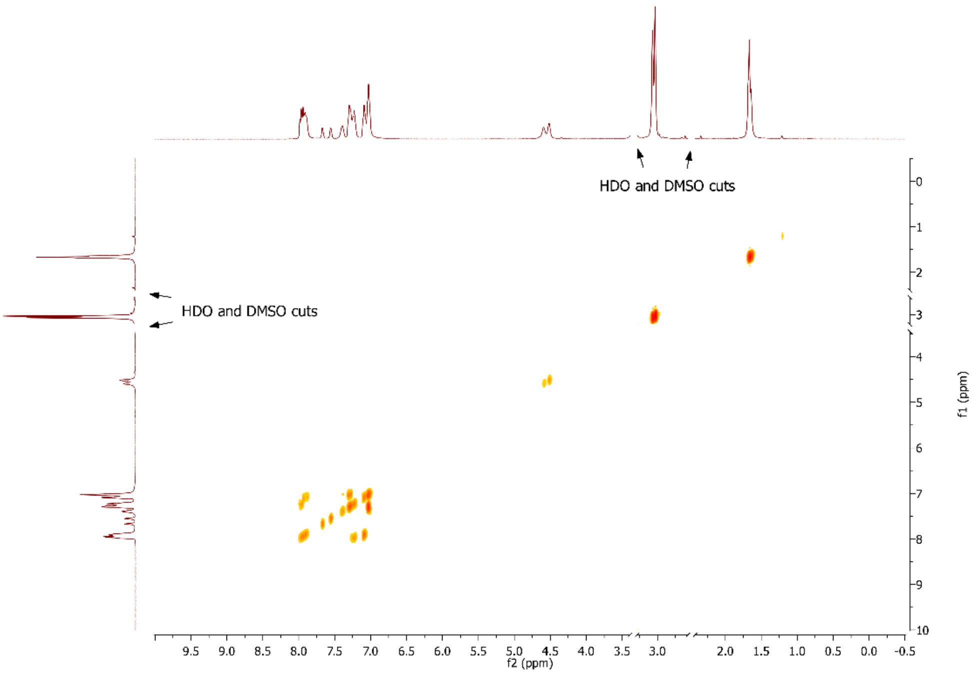

Figure SI3 2D-COSY-NMR PSU100QM

Table S1 – Water uptake, temperature of defunctionalization and decomposition of chloromethylated and quaternarized samples.

SAMPLE	Water loss, %	T_{fg}, °C	T_d, °C
PSU60Cl		329	393
PSU60QM-2	4.8	190	350
PSU60QE-1	4.0	157	350
PSU100Cl		153	348
PSU100QM-1	8.5	151	340
PSU100QE-3	8.2	149	336
PSU150Cl		147	314
PSU150QM-1	9.2	145	308
PSU150QE-2	11.0	138	291
